# The genome sequence of Atlantic Bluefin Tuna, *Thunnus thynnus *(Linnaeus, 1758)

**DOI:** 10.12688/wellcomeopenres.23971.1

**Published:** 2025-03-27

**Authors:** Rebekah A. Oomen, Alessia Cariani, Louise Chavarie, Agostino Leone, Adriana Vella, Noel Vella, Gustav Hellström, Tomas Brodin, Andreas Sundelöf, Mark Blaxter, Ann M. Mc Cartney, Giulio Formenti, Alice Mouton, Fausto Tinti, Fulvio Garibaldi, Petter Lundberg

**Affiliations:** 1 Department of Biological Sciences, University of New Brunswick Saint John, Saint John, New Brunswick, Canada; 2 University of Gothenburg, Gothenburg, Västra Götaland County, Sweden; 3 Centre for Coastal Research, University of Agder, Kristiansand, Norway; 4 University of Oslo Centre for Ecological and Evolutionary Synthesis, Oslo, Oslo, Norway; 5 Department of Biological, Geological & Environmental Science, University of Bologna, Bologna, Italy; 6 Faculty of Environmental Science and Nature Management, Norwegian University of Life Sciences, Ås, Norway; 7 Department of Earth and Marine Sciences (DiSTeM), University of Palermo, Palermo, Italy; 8 National Biodiversity Future Center, Palermo, Italy; 9 Department of Biology, University of Malta, Msida, Malta; 10 Department of Wildlife, Fish and Environmental Studies, Swedish University of Agricultural Sciences, Uppsala, Sweden; 11 Department of Aquatic Resources, Swedish University of Agricultural Sciences, Uppsala, Sweden; 12 Tree of Life, Wellcome Sanger Institute, Hinxton, England, UK; 13 Genomics Institute, University of California Santa Cruz, Santa Cruz, California, USA; 14 School of Biology and Environmental Science, University College Dublin, Dublin, Leinster, Ireland; 15 The Vertebrate Genome Laboratory, The Rockefeller University, New York, New York, USA; 16 Département des sciences et gestion de l'environnement (Arlon Campus Environnement), SEED, University of Liège, Liège, Belgium; 17 Department of Earth, Environment and Life Sciences, University of Genoa, Genova, Italy

**Keywords:** Thunnus thynnus, Atlantic Bluefin Tuna, genome sequence, chromosomal, Scombriformes

## Abstract

We present a genome assembly from a specimen of *Thunnus thynnus* (Atlantic Bluefin Tuna; Chordata; Actinopteri; Scombriformes; Scombridae). The genome sequence has a total length of 799.05 megabases. Most of the assembly (99.17%) is scaffolded into 24 chromosomal pseudomolecules. The mitochondrial genome has also been assembled, with a length of 16.53 kilobases. Gene annotation of this assembly on Ensembl identified 23,266 protein-coding genes.

## Species taxonomy

Eukaryota; Opisthokonta; Metazoa; Eumetazoa; Bilateria; Deuterostomia; Chordata; Craniata; Vertebrata; Gnathostomata; Teleostomi; Euteleostomi; Actinopterygii; Actinopteri; Neopterygii; Teleostei; Osteoglossocephalai; Clupeocephala; Euteleosteomorpha; Neoteleostei; Eurypterygia; Ctenosquamata; Acanthomorphata; Euacanthomorphacea; Percomorphaceae; Pelagiaria; Scombriformes; Scombridae; Scombrinae; Thunnini; *Thunnu*s; *Thunnus thynnus* (Linnaeus, 1758) (NCBI:txid8237)

## Background

The Atlantic bluefin tuna, ( *Thunnus thynnus* [Linnaeus, 1758]; hereafter ‘ *T. thynnus*’) is the largest species within the genus *Thunnus*. *T. thynnus* is distinguished by a reddish-brown second dorsal fin that is higher than the first and short pectoral fins that do not extend to the interspace between the dorsal fins. The lower sides and belly are silvery white, contrasted with faint transverse lines and spots. The anal fin and finlets are bright yellow edged with black and the medial caudal keel is black ( Bariche, 2012; Collette *et al.*, 2021).

*T. thynnus* is a highly migratory, epipelagic, top predator that inhabits the northern Atlantic Ocean and Mediterranean Sea, including the Black Sea. Seasonally, *T. thynnus* approaches closer to shore for spawning, with a western Atlantic component that spawns predominantly in the Gulf of Mexico ( Block *et al.*, 2005; Richardson *et al.*, 2016) and an eastern Atlantic and Mediterranean component that spawns predominantly in the Mediterranean Sea ( Piccinetti *et al.*, 2013).

Stable isotope and genetic studies have revealed weak differentiation between western and eastern components, with the Mediterranean population displaying a panmictic structure ( Antoniou *et al.*, 2017; Díaz-Arce *et al.*, 2024; Puncher *et al.*, 2018; Rodríguez-Ezpeleta *et al.*, 2019; Rooker *et al.*, 2019; Vella *et al.*, 2016), suggesting movement and genetic exchange between the two. The recently discovered spawning ground in the Slope Sea ( Aalto *et al.*, 2023; Hernández *et al.*, 2022; Richardson *et al.*, 2016) exhibits a mix of eastern and western characteristics together with signs of introgression with closely related species ( Díaz-Arce *et al.,* 2024).

Under the generally accepted assumption of natal homing, *T. thynnus* are managed by the International Commission for the Conservation of Atlantic Tunas (ICCAT) as independent eastern and western stocks assigned to discrete spawning areas: Mediterranean Sea and Gulf of Mexico, respectively ( Medina, 2020). The eastern stock undertakes migrations from nutrient-rich summer-autumn feeding grounds in the North Atlantic, as far northward as northern Norway, to oligotrophic winter spawning grounds in the Mediterranean Sea that provide optimal habitat for larval survival ( Malca *et al.*, 2023). The western stock undertakes similar seasonal migrations, feeding as far northward as Newfoundland and Labrador, Canada, and mainly spawning either in the Gulf of Mexico or the Slope Sea. However, transatlantic migrations are not uncommon, particularly westward ( Díaz-Arce *et al.*, 2024).

Beyond the ecological importance of *T. thynnus* as a top predator in Atlantic food webs, they have a long history as an extremely desirable food fish. *T.* thynnu s formed the basis of the world’s oldest commercial fishery ( Di Natale, 2014) and is currently one of the world’s most lucrative fisheries, valued at over one billion USD annually ( Macfadyen *et al.*, 2021). Recreational ‘sport’ fishing of *T. thynnus* is increasingly common where permitted and generates tourism revenue for local communities and funds for supporting conservation research ( Goldsmith *et al.*, 2018; Maar *et al.*, 2022).

Overexploitation of *T. thynnus* led to considerable declines in fishery landings ( MacKenzie *et al.*, 2009; Porch, 2005; Siskey *et al.*, 2016; Vella, 2009), with a number of recent timely management interventions ( ICCAT, 2024) helping populations to recover ( Bjørndal, 2023; Collette *et al.*, 2021). The most recent International Union for Conservation of Nature (IUCN) Red List assessment of *T. thynnus* in 2021 changed the status from “endangered” to “least concern *”* ( Collette *et al.*, 2021). This improvement is typically assigned to more sustainable quotas and successful work against illegal fishing. Despite this global positive trend, there are large regional differences, and the western Atlantic stock is still severely depleted ( Aalto *et al.*, 2021). Recovery may also be affected by climate change, as warmer sea temperatures influence the distribution and abundance of populations ( Erauskin-Extramiana *et al.*, 2019; Fiksen & Reglero, 2022; MacKenzie *et al.*, 2014; Reglero *et al.*, 2018).

This chromosome-level genome assembly will enable accurate alignment of the abundance of population genomic (e.g., RAD-seq and re-sequencing; Antoniou *et al.*, 2017; Díaz-Arce *et al.*, 2024; Puncher *et al.*, 2018; Rodríguez-Ezpeleta *et al.*, 2019) and transcriptomic (e.g. RNA-seq; Marisaldi *et al.*, 2021) data available and planned for this species ( Formenti *et al.*, 2022; Theissinger *et al.*, 2023). These analyses will reveal fine-scale population structure in both single nucleotide polymorphisms and structural variants ( Mérot *et al.*, 2020) across ancient, historical, and contemporary time scales that will improve current management regimes and inform both past and future responses to anthropogenic pressures ( Andrews *et al.*, 2021; Eriksen *et al.*, 2025) as well as potential local adaptation ( Díaz-Arce *et al.*, 2024). Finally, reference-aware RNA-seq analyses will reveal physiological processes potentially differentiating *T. thynnus* from different lineages or with different migratory behaviours or environmental responses ( Oomen & Hutchings, 2017; Theissinger *et al.*, 2023).

## Genome sequence report

### Sequencing data

**Figure 1.**
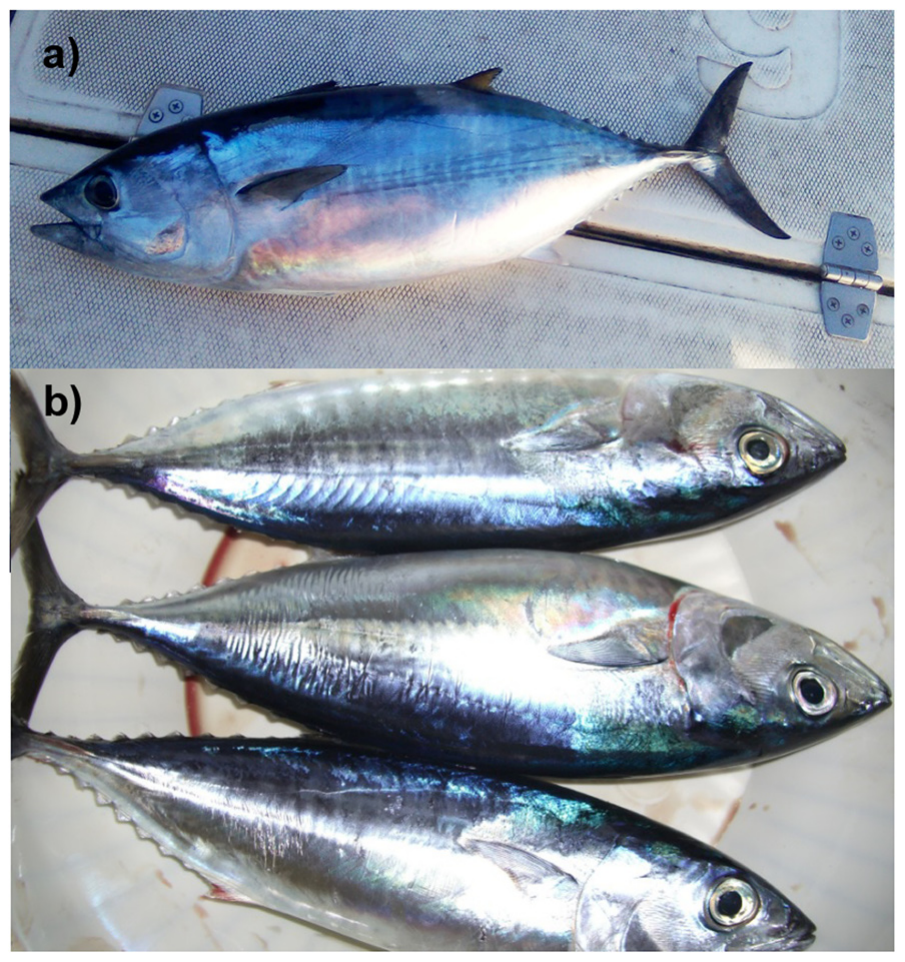
( **a**) Young-of-the-year (YOY) individual measuring approximately 25–30 cm, collected in September 2022 in the Western Mediterranean (Ligurian Sea, Italy). Photograph by Fulvio Garibaldi. ( **b**) YOY individuals measuring approximately 30 cm, collected in September 2013 in the Central Mediterranean (western Ionian Sea, Italy). Photograph by Agostino Leone.

The genome of a specimen of *Thunnus thynnus* ( Figure 1) was sequenced using Pacific Biosciences single-molecule HiFi long reads, generating 30.14 Gb (gigabases) from 5.19 million reads. GenomeScope analysis of the PacBio HiFi data estimated the haploid genome size at 782.63 Mb, with a heterozygosity of 0.75% and repeat content of 10.14%. These values provide an initial assessment of genome complexity and the challenges anticipated during assembly. Based on this estimated genome size, the sequencing data provided approximately 37.0x coverage of the genome. Chromosome conformation Hi-C sequencing produced 145.99 Gb from 966.80 million reads. Table 1 summarises the specimen and sequencing information.

**Table 1.** Specimen and sequencing data for *Thunnus thynnus*.

Project information			
**Study title**	Thunnus thynnus (Atlantic bluefin tuna)		
**Umbrella BioProject**	PRJEB71424		
**Species**	*Thunnus thynnus*		
**BioSpecimen**	SAMEA111406335		
**NCBI taxonomy ID**	8237		
Specimen information			
**Technology**	**ToLID**	**BioSample accession**	**Organism part**
**PacBio long read sequencing**	fThuThy2	SAMEA111406340	muscle
**Hi-C sequencing**	fThuThy3	SAMEA111406341	muscle
Sequencing information			
**Platform**	**Run accession**	**Read count**	**Base count (Gb)**
**Hi-C Illumina NovaSeq 6000**	ERR12512723	9.67e+08	145.99
**PacBio Sequel IIe**	ERR12408775	9.49e+05	5.95
**PacBio Revio**	ERR12408774	4.24e+06	24.19

### Assembly statistics

The primary haplotype was assembled, and contigs corresponding to an alternate haplotype were also deposited in INSDC databases. The assembly was improved by manual curation, which corrected 49 misjoins or missing joins and removed 7 haplotypic duplications. These interventions decreased the scaffold count by 9.77%. The final assembly has a total length of 799.05 Mb in 119 scaffolds, with 468 gaps, and a scaffold N50 of 34.32 Mb ( Table 2).

**Table 2.** Genome assembly data for *Thunnus thynnus*.

Genome assembly		
Assembly name	fThuThy2.1		
Assembly accession	GCA_963924715.1		
*Alternate haplotype accession*	*GCA_963924655.1*		
Assembly level for primary assembly	chromosome		
Span (Mb)	799.05		
Number of contigs	587		
Number of scaffolds	119		
Longest scaffold (Mb)	42.01		
Assembly metric	Measure	*Benchmark*
Contig N50 length	2.96 Mb	*≥ 1 Mb*
Scaffold N50 length	34.32 Mb	*= chromosome N50*
Consensus quality (QV)	Primary: 60.4; alternate: 60.1; combined: 60.3	*≥ 40*
*k*-mer completeness	Primary: 84.08%; alternate: 83.23%; combined: 99.87%	*≥ 95%*
BUSCO *	C:99.2%[S:98.5%,D:0.7%], F:0.3%,M:0.6%,n:3,640	*S > 90%; D < 5%*
Percentage of assembly mapped to chromosomes	99.16%	*≥ 90%*
Sex chromosomes	Not identified	*localised homologous pairs*
Organelles	Mitochondrial genome: 16.53 kb	*complete single alleles*
Genome annotation of assembly GCA_963924715.1 at Ensembl		
Number of protein-coding genes	23,266	
Number of non-coding genes	3,826	
Number of gene transcripts	41,743	

* BUSCO scores based on the actinopterygii_odb10 BUSCO set using version 5.5.0. C = complete [S = single copy, D = duplicated], F = fragmented, M = missing, n = number of orthologues in comparison.

**Figure 2.**
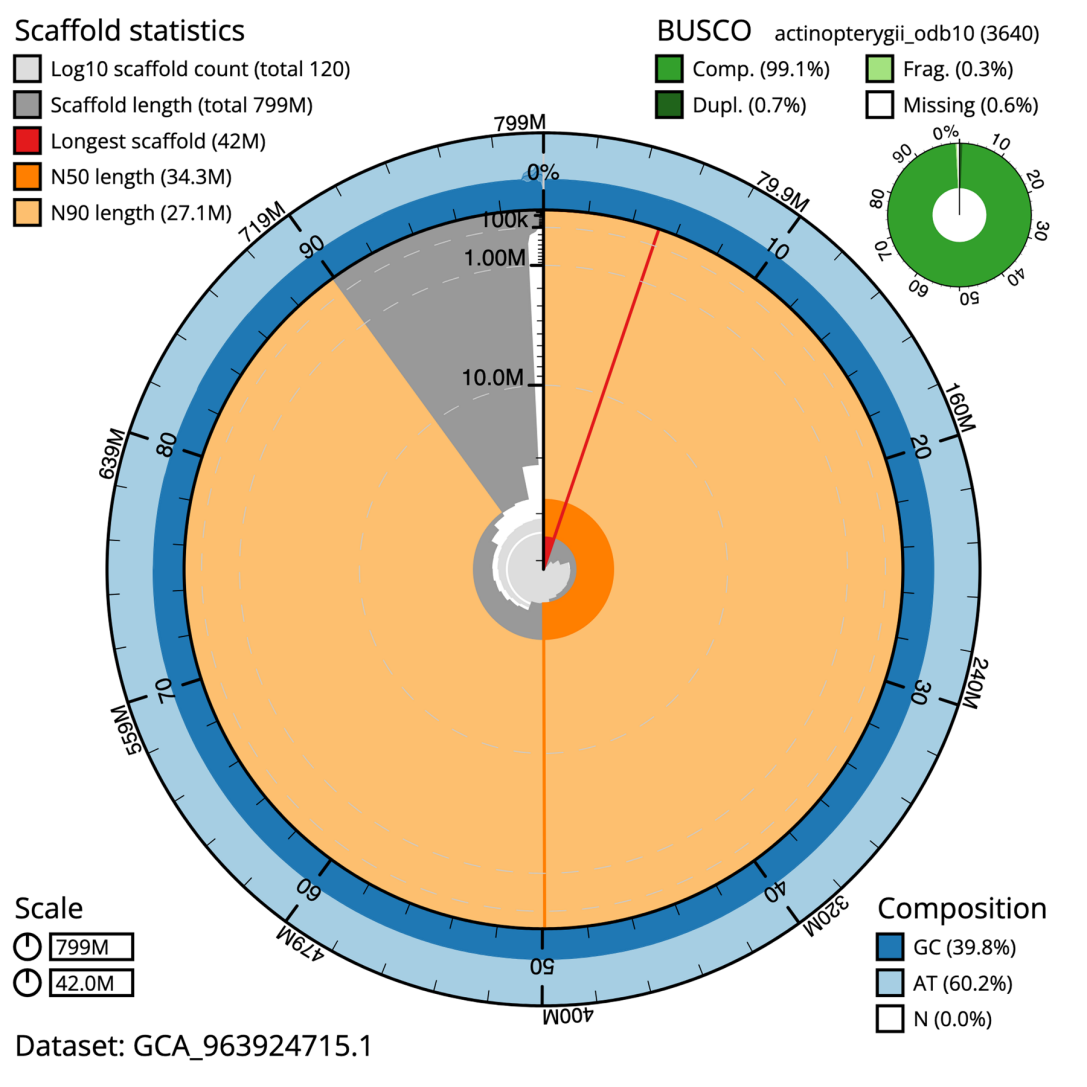
Genome assembly of *Thunnus thynnus*, fThuThy2.1: metrics. The BlobToolKit snail plot provides an overview of assembly metrics and BUSCO gene completeness. The circumference represents the length of the whole genome sequence, and the main plot is divided into 1,000 bins around the circumference. The outermost blue tracks display the distribution of GC, AT, and N percentages across the bins. Scaffolds are arranged clockwise from longest to shortest and are depicted in dark grey. The longest scaffold is indicated by the red arc, and the deeper orange and pale orange arcs represent the N50 and N90 lengths. A light grey spiral at the centre shows the cumulative scaffold count on a logarithmic scale. A summary of complete, fragmented, duplicated, and missing BUSCO genes in the actinopterygii_odb10 set is presented at the top right. An interactive version of this figure is available at https://blobtoolkit.genomehubs.org/view/GCA_963924715.1/dataset/GCA_963924715.1/snail.

**Figure 3.**
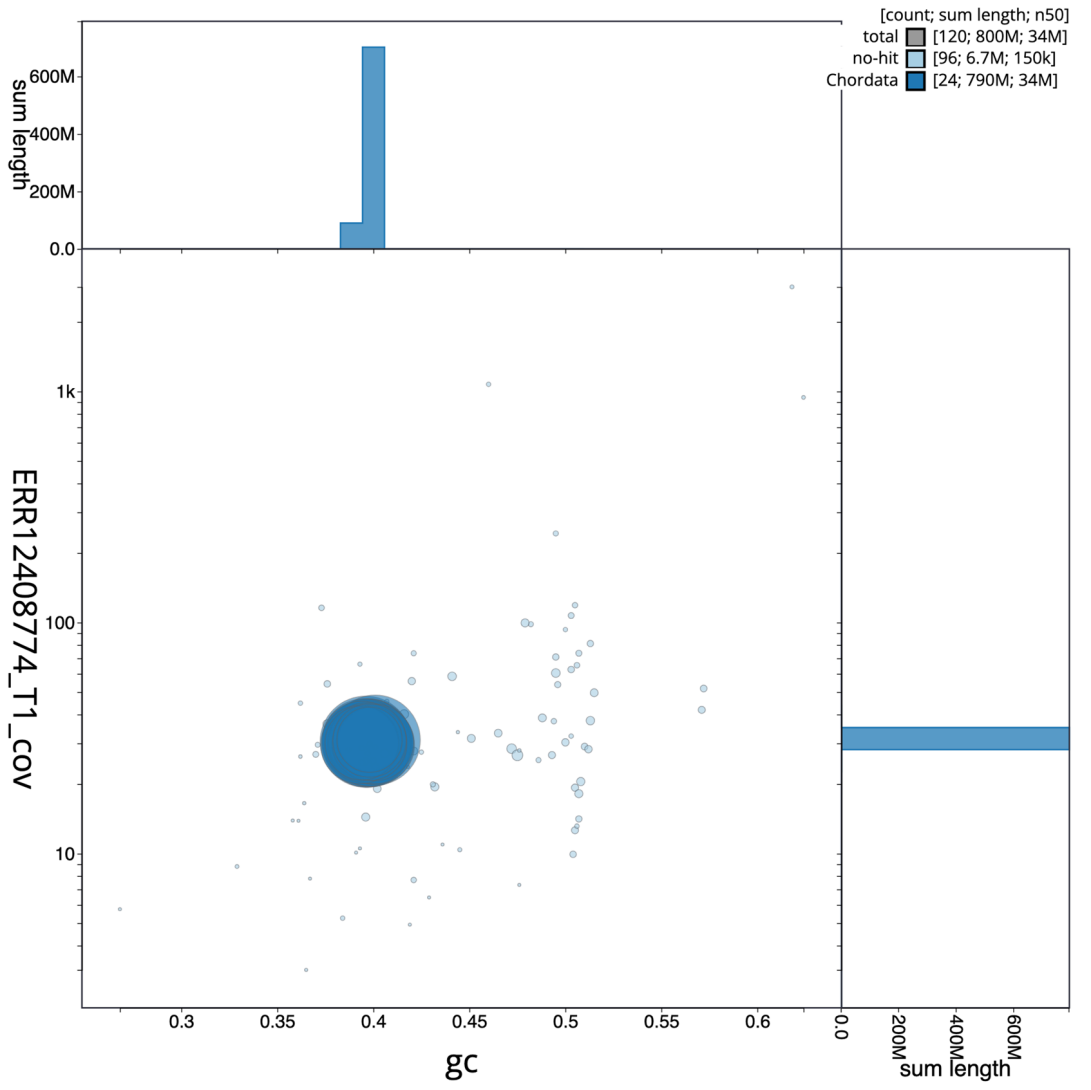
Genome assembly of *Thunnus thynnus*, fThuThy2.1: BlobToolKit GC-coverage plot. Blob plot showing sequence coverage (vertical axis) and GC content (horizontal axis). The circles represent scaffolds, with the size proportional to scaffold length and the colour representing phylum membership. The histograms along the axes display the total length of sequences distributed across different levels of coverage and GC content. An interactive version of this figure is available at https://blobtoolkit.genomehubs.org/view/GCA_963924715.1/dataset/GCA_963924715.1/blob.

**Figure 4.**
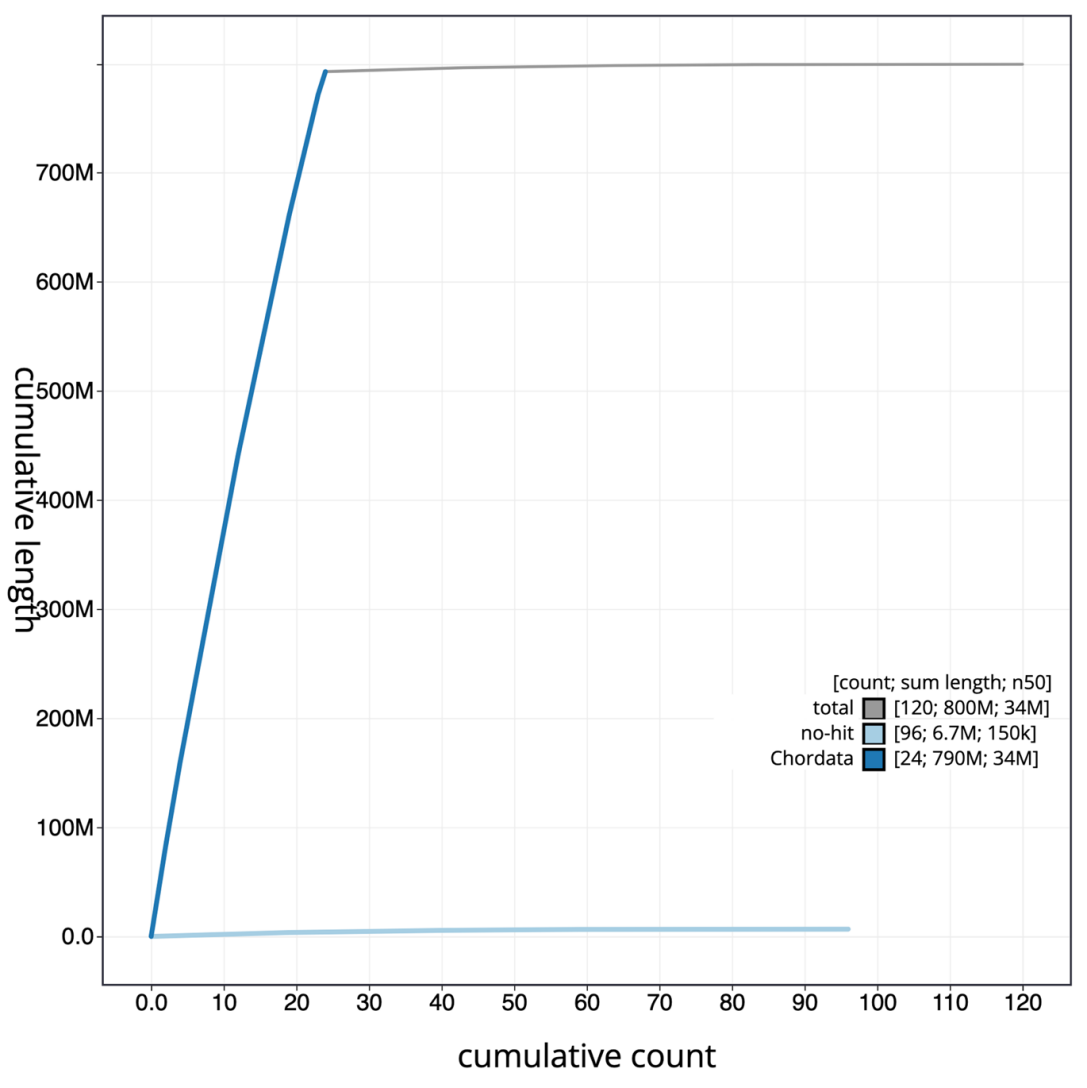
Genome assembly of *Thunnus thynnus,* fThuThy2.1: BlobToolKit cumulative sequence plot. The grey line shows cumulative length for all scaffolds. Coloured lines show cumulative lengths of scaffolds assigned to each phylum using the buscogenes taxrule. An interactive version of this figure is available at https://blobtoolkit.genomehubs.org/view/GCA_963924715.1/dataset/GCA_963924715.1/cumulative.

The snail plot in Figure 2 provides a summary of the assembly statistics, indicating the distribution of scaffold lengths and other assembly metrics. Figure 3 shows the distribution of scaffolds by GC proportion and coverage. Figure 4 presents a cumulative assembly plot, with separate curves representing different scaffold subsets assigned to various phyla, illustrating the completeness of the assembly.

**Figure 5.**
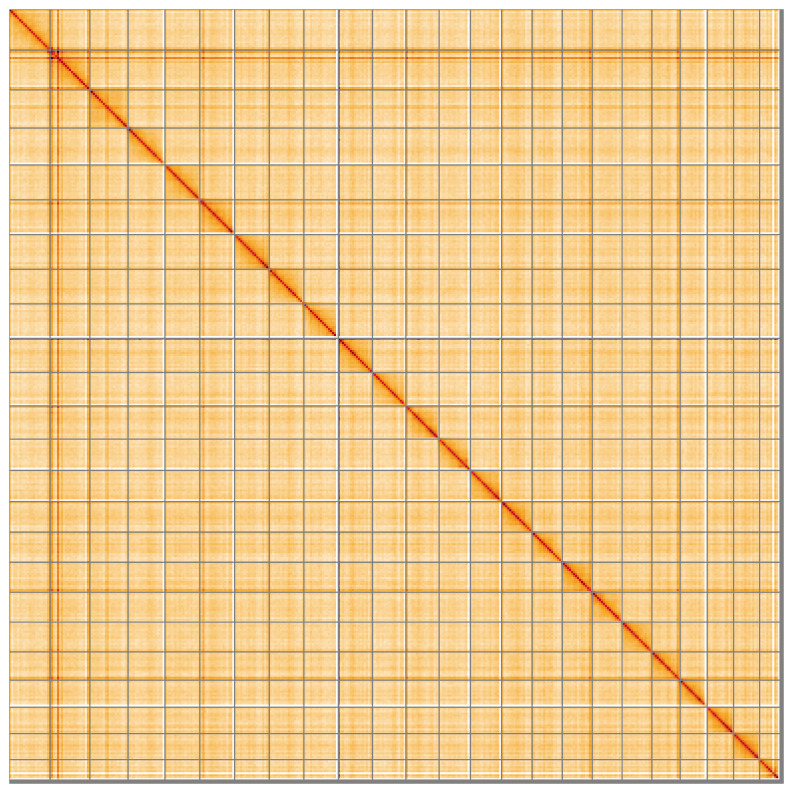
Genome assembly of *Thunnus thynnus:* Hi-C contact map of the fThuThy2.1 assembly, visualised using HiGlass. Chromosomes are shown in order of size from left to right and top to bottom. An interactive version of this figure may be viewed at https://genome-note-higlass.tol.sanger.ac.uk/l/?d=CnETE950TKi3HPqRsxNUKg.

Most of the assembly sequence (99.16%) was assigned to 24 chromosomal-level scaffolds. These chromosome-level scaffolds, confirmed by Hi-C data, are named according to size ( Figure 5; Table 3).

**Table 3.** Chromosomal pseudomolecules in the genome assembly of *Thunnus thynnus*, fThuThy2.

INSDC accession	Name	Length (Mb)	GC%
OZ004732.1	1	42.01	39.5
OZ004733.1	2	40.88	40
OZ004734.1	3	39.3	39.5
OZ004735.1	4	37.85	39.5
OZ004736.1	5	35.84	40
OZ004737.1	6	35.75	40
OZ004738.1	7	35.69	40
OZ004739.1	8	35.55	39.5
OZ004740.1	9	35.45	39.5
OZ004741.1	10	35.06	40
OZ004742.1	11	34.32	39.5
OZ004743.1	12	34.21	39.5
OZ004744.1	13	32.23	39.5
OZ004745.1	14	31.95	39.5
OZ004746.1	15	31.4	39.5
OZ004747.1	16	31.06	40
OZ004748.1	17	30.76	40
OZ004749.1	18	30.67	39.5
OZ004750.1	19	30.62	39.5
OZ004751.1	20	29.11	40
OZ004752.1	21	27.78	40
OZ004753.1	22	27.13	39.5
OZ004754.1	23	26.78	40
OZ004755.1	24	20.98	40
OZ004756.1	MT	0.02	46

The mitochondrial genome was also assembled. This sequence is included as a contig in the multifasta file of the genome submission and as a standalone record.

### Assembly quality metrics

The estimated Quality Value (QV) and *k*-mer completeness metrics, along with BUSCO completeness scores, were calculated for each haplotype and the combined assembly. The QV reflects the base-level accuracy of the assembly, while *k*-mer completeness indicates the proportion of expected *k*-mers identified in the assembly. BUSCO scores provide a measure of completeness based on benchmarking universal single-copy orthologues.

The combined primary and alternate assemblies achieve an estimated QV of 60.3. The *k*-mer recovery for the primary haplotype is 84.08%, and for the alternate haplotype 83.23%; the combined primary and alternate assemblies have a *k*-mer recovery of 99.87%. BUSCO v5.5.0 analysis using the actinopterygii_odb10 reference set ( *n* = 3,640) identified 99.2% of the expected gene set (single = 98.5%, duplicated = 0.7%).

Table 2 provides assembly metric benchmarks adapted from Rhie *et al.* (2021) and the Earth BioGenome Project (EBP) Report on Assembly Standards September 2024. The assembly achieves the EBP reference standard of **6.C.Q60.**

## Genome annotation report

The *Thunnus thynnus* genome assembly (GCA_963924715.1) was annotated at the European Bioinformatics Institute (EBI) on Ensembl Rapid Release. The resulting annotation includes 41,743 transcribed mRNAs from 23,266 protein-coding and 3,826 non-coding genes ( Table 2; https://rapid.ensembl.org/Thunnus_thynnus_GCA_963924715.1/Info/Index). The average transcript length is 18,413.05. There are 1.54 coding transcripts per gene and 10.77 exons per transcript.

## Methods

### Sample acquisition

*Thunnus thynnus* young-of-the-year individual sampling was carried out in Italian waters during scientific activities (tagging and fishing surveys carried out in the Tyrrenian and Ionian Sea during late summer 2013 and summer 2022) following accepted methodologies and under permit. Tissues were sampled and stored in 5 mL ethanol-filled tubes placed in dry ice while onboard. Samples were subsequently stored at –20°C after landing. After 24 hours, samples were shipped in dry ice to the University of Bologna facility where they were stored at -80°C before shipment to the Wellcome Sanger Institute. All specimens were collected and identified by experienced scientists as indicated in the sample manifest. One of the specimens (specimen ID ERGA AV MT 03, ToLID fThuThy2) was used for PacBio DNA sequencing. The specimen used for Illumina Hi-C sequencing was specimen ID ERGA AV MT 06 (ToLID fThuThy2).

### Nucleic acid extraction

The workflow for high molecular weight (HMW) DNA extraction at the Wellcome Sanger Institute (WSI) Tree of Life Core Laboratory includes a sequence of procedures: sample preparation and homogenisation, DNA extraction, fragmentation and purification. Detailed protocols are available on protocols.io ( Denton *et al.*, 2023b). The fThuThy2 sample was prepared for DNA extraction by weighing and dissecting it on dry ice ( Jay *et al.*, 2023). Tissue from the muscle was homogenised using a PowerMasher II tissue disruptor ( Denton *et al.*, 2023a).

HMW DNA was extracted at the WSI Scientific Operations core using the Automated MagAttract v2 protocol ( Oatley *et al.*, 2023). The DNA was sheared into an average fragment size of 12–20 kb in a Megaruptor 3 system ( Bates *et al.*, 2023). Sheared DNA was purified by solid-phase reversible immobilisation, using AMPure PB beads to eliminate shorter fragments and concentrate the DNA ( Strickland *et al.*, 2023). The concentration of the sheared and purified DNA was assessed using a Nanodrop spectrophotometer and Qubit Fluorometer using the Qubit dsDNA High Sensitivity Assay kit. Fragment size distribution was evaluated by running the sample on the FemtoPulse system.

### Hi-C sample preparation

Tissue from the fThuThy3 sample was processed for Hi-C sequencing at the WSI Scientific Operations core, using the Arima-HiC v2 kit. In brief, 20–50 mg of frozen tissue (stored at –80 °C) was fixed, and the DNA crosslinked using a TC buffer with 22% formaldehyde concentration. After crosslinking, the tissue was homogenised using the Diagnocine Power Masher-II and BioMasher-II tubes and pestles. Following the Arima-HiC v2 kit manufacturer's instructions, crosslinked DNA was digested using a restriction enzyme master mix. The 5’-overhangs were filled in and labelled with biotinylated nucleotides and proximally ligated. An overnight incubation was carried out for enzymes to digest remaining proteins and for crosslinks to reverse. A clean up was performed with SPRIselect beads prior to library preparation. Additionally, the biotinylation percentage was estimated using the Qubit Fluorometer v4.0 (Thermo Fisher Scientific) and Qubit HS Assay Kit and Arima-HiC v2 QC beads.

### Library preparation and sequencing

Library preparation and sequencing were performed at the WSI Scientific Operations core.

***PacBio HiFi***

At a minimum, samples were required to have an average fragment size exceeding 8 kb and a total mass over 400 ng to proceed to the low input SMRTbell Prep Kit 3.0 protocol (Pacific Biosciences, California, USA), depending on genome size and sequencing depth required. Libraries were prepared using the SMRTbell Prep Kit 3.0 (Pacific Biosciences, California, USA) as per the manufacturer's instructions. The kit includes the reagents required for end repair/A-tailing, adapter ligation, post-ligation SMRTbell bead cleanup, and nuclease treatment. Following the manufacturer’s instructions, size selection and clean up was carried out using diluted AMPure PB beads (Pacific Biosciences, California, USA). DNA concentration was quantified using the Qubit Fluorometer v4.0 (Thermo Fisher Scientific) with Qubit 1X dsDNA HS assay kit and the final library fragment size analysis was carried out using the Agilent Femto Pulse Automated Pulsed Field CE Instrument (Agilent Technologies) and gDNA 55kb BAC analysis kit.

Samples were sequenced using both the Sequel IIe system and the Revio instrument (Pacific Biosciences, California, USA). The concentration of the library loaded onto the Sequel IIe was in the range 40–135 pM. Samples were sequenced on a Revio instrument (Pacific Biosciences, California, USA). Prepared libraries were normalised to 2 nM, and 15 μL was used for making complexes. Primers were annealed and polymerases were hybridised to create circularised complexes according to manufacturer’s instructions. The complexes were purified with the 1.2X clean up with SMRTbell beads. The purified complexes were then diluted to the Revio loading concentration (in the range 200–300 pM), and spiked with a Revio sequencing internal control. Samples were sequenced on Revio 25M SMRT cells (Pacific Biosciences, California, USA). The SMRT link software, a PacBio web-based end-to-end workflow manager, was used to set-up and monitor the sequencing runs, as well as perform primary and secondary analysis of the data upon completion.

***Hi-C***

For Hi-C library preparation, biotinylated DNA constructs were fragmented using the Covaris E220 sonicator and size selected using SPRISelect beads to 400 to 600 bp using the INTEGRA MINI 96 (INTEGRA). The DNA was then enriched using the Arima-HiC v2 kit Enrichment beads on the INTEGRA MINI 96 (INTEGRA). The NEBNext Ultra II DNA Library Prep Kit (New England Biolabs) was used for end repair, A-tailing, and adapter ligation. This uses a custom protocol which resembles the standard NEBNext Ultra II DNA Library Prep protocol but where library preparation occurs while DNA is bound to the Enrichment beads. Library PCR amplification was carried out using KAPA HiFi Hot start mix and a custom IDT UDI (Unique Dual Index) 96 barcode plate (Integrated DNA Technologies). Depending on sample concentration and biotinylation percentage determined at the crosslinking stage, samples were run for 10-16 PCR cycles. Post-PCR, samples were cleaned-up using SPRISelect beads and the INTEGRA MINI 96 (INTEGRA). The Hi-C sequencing was performed using paired-end sequencing with a read length of 150 bp on an Illumina NovaSeq 6000 instrument.

### Genome assembly, curation and evaluation

***Assembly***

Prior to assembly of the PacBio HiFi reads, a database of *k*-mer counts ( *k* = 31) was generated from the filtered reads using FastK. GenomeScope2 ( Ranallo-Benavidez *et al.*, 2020) was used to analyse the *k*-mer frequency distributions, providing estimates of genome size, heterozygosity, and repeat content.

The HiFi reads were first assembled using Hifiasm ( Cheng *et al.*, 2021) with the --primary option. Haplotypic duplications were identified and removed using purge_dups ( Guan *et al.*, 2020). The Hi-C reads were mapped to the primary contigs using bwa-mem2 ( Vasimuddin *et al.*, 2019). The contigs were further scaffolded using the provided Hi-C data ( Rao *et al.*, 2014) in YaHS ( Zhou *et al.*, 2023) using the --break option for handling potential misassemblies. The scaffolded assemblies were evaluated using Gfastats ( Formenti *et al.*, 2022), BUSCO ( Manni *et al.*, 2021) and MERQURY.FK ( Rhie *et al.*, 2020).

The mitochondrial genome was assembled using MitoHiFi ( Uliano-Silva *et al.*, 2023), which runs MitoFinder ( Allio *et al.*, 2020) and uses these annotations to select the final mitochondrial contig and to ensure the general quality of the sequence.

***Assembly curation***

The assembly was decontaminated using the Assembly Screen for Cobionts and Contaminants (ASCC) pipeline. Flat files and maps used in curation were generated via the TreeVal pipeline ( Pointon *et al.*, 2023). Manual curation was conducted primarily in PretextView ( Harry, 2022) and HiGlass ( Kerpedjiev *et al.*, 2018), with additional insights provided by JBrowse2 ( Diesh *et al.*, 2023). Scaffolds were visually inspected and corrected as described by Howe *et al*. (2021). Any identified contamination, missed joins, and mis-joins were amended, and duplicate sequences were tagged and removed. The curation process is documented at https://gitlab.com/wtsi-grit/rapid-curation.

***Assembly quality assessment***

The Merqury.FK tool ( Rhie *et al.*, 2020), run in a Singularity container ( Kurtzer *et al.*, 2017), was used to evaluate *k*-mer completeness and assembly quality for the primary and alternate haplotypes using the *k*-mer databases ( *k* = 31) that were computed prior to genome assembly. The analysis outputs included assembly QV scores and completeness statistics.

A Hi-C contact map was produced for the final version of the assembly. The Hi-C reads were aligned using bwa-mem2 ( Vasimuddin *et al.*, 2019) and the alignment files were combined using SAMtools ( Danecek *et al.*, 2021). The Hi-C alignments were converted into a contact map using BEDTools ( Quinlan & Hall, 2010) and the Cooler tool suite ( Abdennur & Mirny, 2020). The contact map was visualised in HiGlass ( Kerpedjiev *et al.*, 2018).

The blobtoolkit pipeline is a Nextflow port of the previous Snakemake Blobtoolkit pipeline ( Challis *et al.*, 2020). It aligns the PacBio reads in SAMtools and minimap2 ( Li, 2018) and generates coverage tracks for regions of fixed size. In parallel, it queries the GoaT database ( Challis *et al.*, 2023) to identify all matching BUSCO lineages to run BUSCO ( Manni *et al.*, 2021). For the three domain-level BUSCO lineages, the pipeline aligns the BUSCO genes to the UniProt Reference Proteomes database ( Bateman *et al.*, 2023) with DIAMOND blastp ( Buchfink *et al.*, 2021). The genome is also divided into chunks according to the density of the BUSCO genes from the closest taxonomic lineage, and each chunk is aligned to the UniProt Reference Proteomes database using DIAMOND blastx. Genome sequences without a hit are chunked using seqtk and aligned to the NT database with blastn ( Altschul *et al.*, 1990). The blobtools suite combines all these outputs into a blobdir for visualisation.

The blobtoolkit pipeline was developed using nf-core tooling ( Ewels *et al.*, 2020) and MultiQC ( Ewels *et al.*, 2016), relying on the Conda package manager, the Bioconda initiative ( Grüning *et al.*, 2018), the Biocontainers infrastructure ( da Veiga Leprevost *et al.*, 2017), as well as the Docker ( Merkel, 2014) and Singularity ( Kurtzer *et al.*, 2017) containerisation solutions.

Table 4 contains a list of relevant software tool versions and sources.

**Table 4.** Software tools: versions and sources.

Software tool	Version	Source
BEDTools	2.30.0	https://github.com/arq5x/bedtools2
BLAST	2.14.0	ftp://ftp.ncbi.nlm.nih.gov/blast/executables/blast+/
BlobToolKit	4.3.3	https://github.com/blobtoolkit/blobtoolkit
BUSCO	5.5.0	https://gitlab.com/ezlab/busco
bwa-mem2	2.2.1	https://github.com/bwa-mem2/bwa-mem2
Cooler	0.8.11	https://github.com/open2c/cooler
DIAMOND	2.1.8	https://github.com/bbuchfink/diamond
fasta_windows	0.2.4	https://github.com/tolkit/fasta_windows
FastK	666652151335353eef2fcd58880bcef5bc2928e1	https://github.com/thegenemyers/FASTK
Gfastats	1.3.6	https://github.com/vgl-hub/gfastats
GoaT CLI	0.2.5	https://github.com/genomehubs/goat-cli
Hifiasm	0.19.5-r587	https://github.com/chhylp123/hifiasm
HiGlass	44086069ee7d4d3f6f3f0012569789ec138f42b84 aa44357826c0b6753eb28de	https://github.com/higlass/higlass
MerquryFK	d00d98157618f4e8d1a9190026b19b471055b22e	https://github.com/thegenemyers/MERQURY.FK
Minimap2	2.24-r1122	https://github.com/lh3/minimap2
MitoHiFi	3	https://github.com/marcelauliano/MitoHiFi
MultiQC	1.14, 1.17, and 1.18	https://github.com/MultiQC/MultiQC
Nextflow	23.04.1	https://github.com/nextflow-io/nextflow
PretextView	0.2.5	https://github.com/sanger-tol/PretextView
purge_dups	1.2.5	https://github.com/dfguan/purge_dups
samtools	1.18	https://github.com/samtools/samtools
sanger-tol/ ascc	-	https://github.com/sanger-tol/ascc
sanger-tol/ blobtoolkit	0.3.0	https://github.com/sanger-tol/blobtoolkit
Seqtk	1.3	https://github.com/lh3/seqtk
Singularity	3.9.0	https://github.com/sylabs/singularity
TreeVal	1.2.0	https://github.com/sanger-tol/treeval
YaHS	1.2a.2	https://github.com/c-zhou/yahs

### Genome annotation

The Ensembl Genebuild annotation system ( Aken *et al.*, 2016) was used to generate annotation for the *Thunnus thynnus* assembly (GCA_963924715.1) in Ensembl Rapid Release at the EBI. Annotation was created primarily through alignment of transcriptomic data to the genome, with gap filling via protein-to-genome alignments of a select set of proteins from UniProt ( UniProt Consortium, 2019).

### Wellcome Sanger Institute – Legal and Governance

The materials that have contributed to this genome note have been supplied by a Tree of Life collaborator. The Wellcome Sanger Institute employs a process whereby due diligence is carried out proportionate to the nature of the materials themselves, and the circumstances under which they have been/are to be collected and provided for use. The purpose of this is to address and mitigate any potential legal and/or ethical implications of receipt and use of the materials as part of the research project, and to ensure that in doing so we align with best practice wherever possible.

The overarching areas of consideration are:

•   Ethical review of provenance and sourcing of the material

•   Legality of collection, transfer and use (national and international)

Each transfer of samples is undertaken according to a Research Collaboration Agreement or Material Transfer Agreement entered into by the Tree of Life collaborator, Genome Research Limited (operating as the Wellcome Sanger Institute) and in some circumstances other Tree of Life collaborators.

## Data Availability

European Nucleotide Archive: Thunnus thynnus (Atlantic bluefin tuna). Accession number PRJEB71424; https://identifiers.org/ena.embl/PRJEB71424. The genome sequence is released openly for reuse. The *Thunnus thynnus* genome sequencing initiative is part of the European Reference Genome Atlas Pilot Project (PRJEB71424) and the Vertebrate Genomes Project (PRJNA489243). All raw sequence data and the assembly have been deposited in INSDC databases. Raw data and assembly accession identifiers are reported in Table 1 and Table 2.

## Author information

Members of the Wellcome Sanger Institute Tree of Life Management, Samples and Laboratory team are listed here: https://doi.org/10.5281/zenodo.12162482.

Members of Wellcome Sanger Institute Scientific Operations: Sequencing Operations are listed here: https://doi.org/10.5281/zenodo.12165051.

Members of the Wellcome Sanger Institute Tree of Life Core Informatics team are listed here: https://doi.org/10.5281/zenodo.12160324.

Members of the Tree of Life Core Informatics collective are listed here: https://doi.org/10.5281/zenodo.12205391.
