# Supplementary material for: The genome sequence of Atlantic Bluefin Tuna, Thunnus thynnus (Linnaeus, 1758)
Source: Wellcome Open Res. 2025 Mar 27;10:163. [Version 1] doi: 10.12688/wellcomeopenres.23971.1 (PMC12186022; doi:10.12688/wellcomeopenres.23971.1)
Supplement: Peer review history

# Reviewer report: Reviewer response for version 1

**DOI:** 10.21956/wellcomeopenres.26447.r121525

**Published:** 2025-06-23

**Authors:** Fabiano B. Menegidio

**Affiliations:** 1 University of Mogi das Cruzes (UMC), Mogi das Cruzes, Brazil

The article presents a high-quality genome assembly of the Atlantic bluefin tuna ( *Thunnus thynnus*), comprising 799.05 Mb organized into 119 scaffolds, with 99.16% of the sequence allocated to 24 pseudomolecules, in addition to the complete assembly of the mitochondrial genome (16.53 kb). Sequencing was performed using PacBio HiFi technology, complemented by Illumina Hi-C data for chromosome-level scaffolding. The assembly meets the EBP 6.C.Q60 standard of excellence, with a consensus quality value (QV) ≥ 60, k-mer completeness of 99.87%, and 99.2% BUSCO completeness using the actinopterygii_odb10 dataset. Annotation, carried out through Ensembl Rapid Release, identified 23,266 protein-coding genes and 3,826 non-coding genes, distributed across more than 41,000 transcripts.

 The protocols employed are technically appropriate and reflect the current state of the art in vertebrate genomics, with up-to-date tools (Hifiasm, YaHS, BUSCO, MERQURY, MitoHiFi, among others), rigorous assembly validation, and manual curation using HiGlass and PretextView. The methods are sufficiently described to enable replication, particularly with the support of publicly available detailed protocols through protocols.io. The datasets are accessible, well-documented, and provided in widely interoperable formats, with clear metadata and persistent identifiers in ENA (PRJEB71424) and Ensembl (GCA_963924715.1).

 The main limitation of the manuscript lies in the absence of a clearly structured justification for the generation of the dataset. While the ecological, economic, and conservation importance of the species is mentioned, the authors do not explicitly present the scientific motivations for producing a new reference genome—such as gaps in previous assemblies, the need for chromosome-level data for population management, or alignment with initiatives like ERGA or the VGP. It is strongly recommended that the authors include a specific paragraph clearly stating the scientific relevance and objectives of this resource, as expected in data-oriented publications.

 Additionally, the authors are encouraged to provide a more complete description of the mitochondrial genome, which was successfully assembled and referenced in the text but not presented in detail. The mitogenome is an essential resource for phylogenetic, population, and biodiversity studies, and it is standard practice to include its gene structure, a circular genome map, an annotation table, and ideally, basic comparisons with mitogenomes from other populations or *Thunnus* species. Including this section would reinforce the completeness of the study and enhance its value to the scientific community.

 Other suggestions to strengthen the manuscript include: (i) presenting a visual flowchart of the assembly and annotation pipeline; (ii) detailing key parameters used in bioinformatics tools (such as Hifiasm, YaHS, and purge_dups); (iii) explicitly stating the license under which the data are made available (e.g., CC BY 4.0 in public repositories); and (iv) clarifying whether the sequenced specimen represents a specific geographic lineage, considering the known population structure of the species.

 In conclusion, the work is technically robust, with high-quality, well-documented, and reproducible data. The inclusion of a clear scientific justification and the formal presentation of the mitochondrial genome are the two main points that should be addressed for the article to fully meet the standards of a data-driven publication.

Are sufficient details of methods and materials provided to allow replication by others?

Yes

Is the rationale for creating the dataset(s) clearly described?

Partly

Are the datasets clearly presented in a useable and accessible format?

Yes

Are the protocols appropriate and is the work technically sound?

Yes

Reviewer Expertise:

Omics Sciences and Bioinformatics

I confirm that I have read this submission and believe that I have an appropriate level of expertise to confirm that it is of an acceptable scientific standard.

# Reviewer report: Reviewer response for version 1

**DOI:** 10.21956/wellcomeopenres.26447.r123700

**Published:** 2025-06-12

**Authors:** Kathryn Elmer

**Affiliations:** 1 University of Glasgow, Scotland, UK

The authors have prepared a brief note on the reference genome development. This includes a short background of the relevance of the species for fisheries and commercial interests as well as the scientific aspects.

 The analyses are state of the art and the resulting genome is outstanding BUSCO completeness. The genome will provide an important contribution to the scientific community

 The methods are generally well described. I agree with the other reviewers about some points that are a bit unclear.

 Can the geographic origins not be more precise, especially since two different samples at such different time periods were used?

 Which tissues were involved is not clear.

 Small thing, but there is no need to say “hereafter ‘T. thynnus.’ since such abbreviation of genus name is standard

 Information on RNA and RNAseq methods seems to be missing though it seems to have been generated. MS notes “Annotation was created primarily through alignment of transcriptomic data to the genome”

Are sufficient details of methods and materials provided to allow replication by others?

Partly

Is the rationale for creating the dataset(s) clearly described?

Yes

Are the datasets clearly presented in a useable and accessible format?

Yes

Are the protocols appropriate and is the work technically sound?

Yes

Reviewer Expertise:

evolutionary biology

I confirm that I have read this submission and believe that I have an appropriate level of expertise to confirm that it is of an acceptable scientific standard, however I have significant reservations, as outlined above.

# Reviewer report: Reviewer response for version 1

**DOI:** 10.21956/wellcomeopenres.26447.r123703

**Published:** 2025-06-03

**Authors:** Steven M Mussmann

**Affiliations:** 1 Southwestern Native Aquatic Resources and Recovery Center, Dexter, USA

Blaxter et al. report on the assembly of a reference genome for the Atlantic Bluefin Tuna Thunnus thynnus. They provide a brief background on the life history, distribution, population structure, and management of this commercially important species. They also describe the methods for assembling the genome into 24 chromosomal pseudo-molecules from PacBio HiFi data and short-read Hi-C data. The authors provide good justification for undertaking this work. They state that this reference genome was assembled for the purpose of aligning population genomic data and transcriptome data to address many research questions including fine-scale population structure, physiological processes, fisheries management, and others.

 The authors provide a great amount of detail on most lab and bioinformatic methods. In general I think someone could replicate the work done here from their descriptions. However, the description of annotation methods was much little less detailed than other sections (this is why I answered 'partly' to "Are sufficient details of methods and materials provided to allow replication by others?"). They also do not mention sequencing RNA that I can find, so I suspect this was *ab initio* gene prediction? or mRNA reads were obtained from another source? I also think it would be advantageous if the authors could post their scripts written to conduct the genome assembly procedures on a public site such as Github. This would be a great supplement to the raw data and the assembly which are already available in public databases. Overall I think this data note is exceptionally well put together, and I have just a few additional minor suggestions which are provided below.

 Minor comments:

 ● This is a really minor thing, but historically fish images have been displayed in left lateral view in scientific publications. The photo in figure 1b would better conform to this standard if it was rotated 180 degrees so that the two fish currently on top have their heads pointing to the left.

 ● The authors mention a number of manual edits to the initial scaffolds assembled from the Hi-C data. They provide a contact map of the final product, but it could be beneficial to provide a before/after version for readers to see.

Are sufficient details of methods and materials provided to allow replication by others?

Partly

Is the rationale for creating the dataset(s) clearly described?

Yes

Are the datasets clearly presented in a useable and accessible format?

Yes

Are the protocols appropriate and is the work technically sound?

Yes

Reviewer Expertise:

Genomics, population genetics, bioinformatics, applied conservation genetics

I confirm that I have read this submission and believe that I have an appropriate level of expertise to confirm that it is of an acceptable scientific standard, however I have significant reservations, as outlined above.

# Reviewer report: Reviewer response for version 1

**DOI:** 10.21956/wellcomeopenres.26447.r122012

**Published:** 2025-05-10

**Authors:** Richard Coleman, Matilde Lanzini

**Affiliations:** 1 Marine Biology and Ecology, University of Miami Rosenstiel School of Marine and Atmospheric Science, Miami, Florida, USA

This manuscript presents genome assembly for the Atlantic Bluefin Tuna ( *Thunnus* *thynnus*). The authors have done a great job detailing the process with thorough descriptions of each step. I have identified several areas that can be addressed to improve clarity prior to indexing.

Methods

- Tyrrenian should be Tyrrhenian
- Throughout the methods section, terms such as tissues and specimens are used prior to indicating what the total sample size is, which is never described.
- The sample names (described below) also add to this confusion. Are there only two specimens used, one for PacBio and the other for Illumina Hi-C? Also, what type of tissue is used for sequencing?
- What is the concentration of the ethanol that is used for tissue preservation?
- “The fThuThy2 sample was prepared for DNA extraction...”. Both samples have fThuThy2 in their name so it is unclear which sample is being referred to here.

Hi-C sample preparation

- “Tissue from the fThuThy3 sample was processed...” The previous section indicated that ID ERGA AV MT 06 (ToLID fThuThy2) was the sample used for Hi-C sequencing. Additionally, Table 1 lists the Hi-C samples as fThuThys3. Please confirm the sample names between sections.
- “DNA was digested using a restriction enzyme master mix.” Please provide details of the specific enzymes used as part of the digestion process.
- Figure 5 – what are the axes for this figure? Where is chromosome 1?

Are sufficient details of methods and materials provided to allow replication by others?

Partly

Is the rationale for creating the dataset(s) clearly described?

Yes

Are the datasets clearly presented in a useable and accessible format?

Yes

Are the protocols appropriate and is the work technically sound?

Yes

Reviewer Expertise:

Molecular ecology, population genetics

We confirm that we have read this submission and believe that we have an appropriate level of expertise to confirm that it is of an acceptable scientific standard, however we have significant reservations, as outlined above.
